# Cranial Functional Movement Disorders: A Case Series with Literature Review

**DOI:** 10.5334/tohm.352

**Published:** 2020-08-25

**Authors:** Anumeha Mishra, Sanjay Pandey

**Affiliations:** 1 Department of Neurology, Govind Ballabh Pant Institute of Postgraduate Medical Education and Research, JLN Marg, New Delhi, IN

**Keywords:** Cranial, Functional, Hemifacial spasm

## Abstract

**Background:**

Cranial functional movement disorders (CFMDs) affect the face, eyes, jaw, tongue, and palate.

**Objectives:**

We aimed to examine our large series of functional movement disorders (FMDs) patients where the cranial muscles were involved to determine their phenomenology and other clinical features.

**Methods:**

This is a chart review of 26 patients who presented with CFMDs.

**Results:**

There were 16 (61.53%) females and 10 (38.46%) male patients. The mean ± [standard deviation (SD)] age at the presentation was 33.96 ± 16.94 (Range: 11–83) years. The duration of symptoms ranged from one day to 6 years (Mean ±SD: 402.03 ±534.97 days). According to the Fahn-Williams criteria, CFMDs were documented in 24 patients and clinically established in two patients. The facial [38.46% (10/26)] involvement was the most common in our CFMDs patients. Oromandibular [19.23% (5/26)], ocular [15.38% (4/26)], lingual [15.38% (4/26)], speech [15.38% (4/26)] and palatal [(3.85; 1/26)] involvement was also seen. 10 (38.46%) patients also had associated FMD in the extracranial regions. Precipitating factors were present in 84.61% (22/26) of the patients and associated illnesses were present in 42.30% (11/26) of the patients. At 3 months follow-up, 9 (34.61%) patients had improved, 13 (50%) had partial improvement and 4 (15.38%) had no improvement.

**Conclusions:**

There was a slight female preponderance in our patients. CFMDs are more likely to involve facial muscles. Associated medical conditions like neuropsychiatric disturbances and headaches are frequently present in CFMDs patients. Early clinical diagnosis will avoid unnecessary investigations and allow the patient to seek the right treatment.

## Introduction

The intriguing history of cranial functional movement disorders (CFMDs) dates back to the era of Jean-Martin Charcot in 1887 when on the first examination he had mistaken a patient with left-sided functional facial spasm for that of an organic facial palsy of the lower right half of the face [1]. Later on, he went on to publish various drawings of similarly afflicted patients in his journal. A pupil of Charcot, Tourette, in 1889, described hysterical blepharospasm in the same journal [2]. Gowers beautifully described the quivering movements of the orbicularis that did not resemble a true facial spasm, mentioned about ‘wrong way’ tongue deviation wherein the tongue would deviate to the side of facial spasm which is the opposite of what would be expected in a true pontomedullary lesion, and was perspicacious in his observation that these movements usually lessened by physical and mental rest and almost always increased by emotion, and by the movement of the face [3]. Babinski with Froment and then Dejerine wrote about hysteric ‘glossolabial spasm’ [4,5]. Casey Wood in 1898 in his essay titled ‘The Detection of Hysteria’ from an ophthalmological perspective wrote, ‘if one were to make a special study of that organ that most uniformly exhibit the evidence of hysteria, the eye would afford the most information, even more emphatically than the skin or the mucous membranes [6].

In the last decade, there are some important case series published regarding the description of cranial functional movement disorders (CFMDs) [7,8,9]. But still, the data remains scarce and cranial movement disorders affecting the eyes, tongue or other facial muscles are often an under-recognized feature of patients with functional movement disorders (FMDs).

In this study, we aimed to examine FMDs patients where the cranial muscles were involved to determine their phenomenology, precipitating factors, and associated co-morbidities and do a systematic review of the published literature.

## Patients and methods

**Figure 1:**
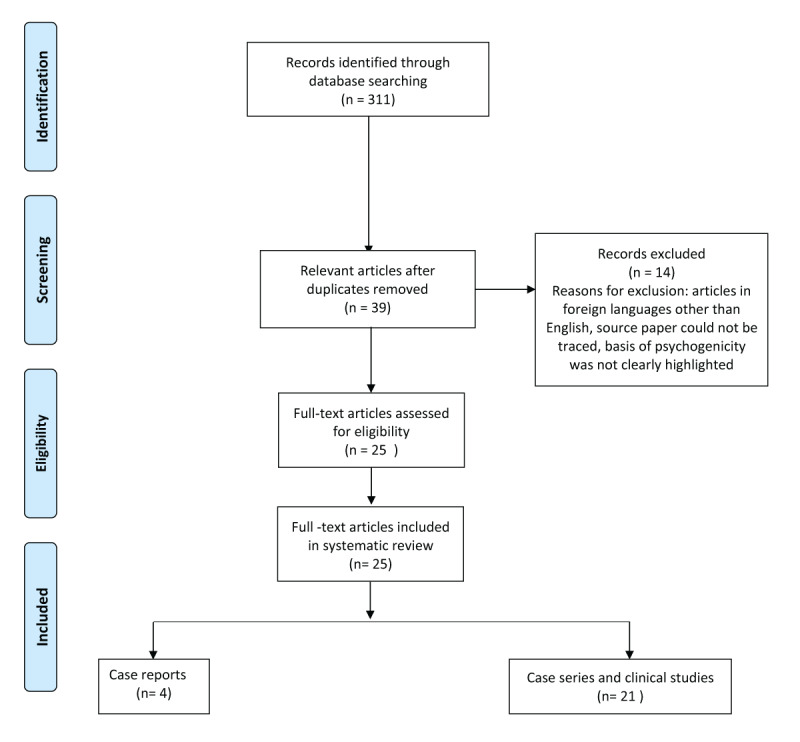
Search strategy for the systematic review of cranial functional movement disorders.

This is a detailed chart review of 26 consecutive patients with CFMDs who attended our movement disorder clinic over the past 2 years and were diagnosed using the Fahn and Williams criteria [10]. This retrospective chart review was done under our study on ‘Functional Neurological Disorder Patients’ approved by the institutional ethics committee (IEC) of Maulana Azad Medical College and associated hospitals. Written informed consent was obtained from all participating individuals according to the IEC guidelines. We also analyzed the videos of the patient to look for details regarding the phenomenology of the movement disorders. In addition to demographic details, data were also collected regarding the phenomenology, antecedent illnesses, precipitating factors, treatment received, and outcome. We made all efforts to contact the referring physician to get the previous medical records. All patients had received treatment with counselling, cognitive behavioural therapy, and pharmacotherapy with consultation from the Psychiatry department. The outcome was categorized as improved, partially improved, and not improved at the end of three months. Patients who initially improved but had frequent relapses with a variable course during the three months were classified as partially improved. We also completed a PubMed search (Figure 1), on 28^th^ March 2020, using the keywords ‘Cranial functional movement disorders’ and ‘Cranial psychogenic movement disorder’. Out of 311 articles that were retrieved, 25 relevant articles were included in our literature review.

The data was entered in the Microsoft office excel sheet and analysed using the SPSS software version 18. Values were expressed as mean ± standard deviations (SD) and as percentages and ranges. Frequencies between the various groups were compared using the χ^2^ test, and *p* values ≤ 0.05 were considered statistically significant.

## Results: (Tables 1 and 2)

**Table 1:** Demographic and clinical characteristics of the cranial functional movement disorders patients.

	Demographic and clinical characteristics (n = 26)	Findings

1.	AGE	Mean ± standard deviation (SD): 33.96 ± 16.94 (Range: 11 to 83) years
	Children (age of onset <18 years)	5 (19.23%)
	Adults (18–60 years)	19 (73.07%)
	Elderly (age of onset >60 years)	2 (7.69%)
2.	MALE: FEMALE	10:16
3.	DURATION	Mean ± SD (402.03 ± 534.97), Range: 1 day–6 years
4.	ONSET	
	Abrupt: 7 (26.92%)	Gradual: 19 (73.07%)
5.	LEVEL OF CERTAINTY	
	Documented: 24 (92.30%)	Clinically established: 2 (7.69%)
6.	PHENOMENOLOGY	
	***Ocular/peri-ocular:* 4 (15.38%)** Blepharospasm: 1 (3.8%) Ptosis 1 (3.8%) Eyelid myoclonus: 1 (3.8%) Eyebrow dyskinesia: 1 (3.8%)	***Facial:* 10 (38.46%)** Hemifacial spasm: 4 (15.38%) Orofacial dyskinesia: 5 (19.23%) Pure facial dyskinesia: 1 (3.8%)
	*Oromandibular*: 5 (19.23%) *Lingual*: 4 (15.38%)	*Palatal*: 1 (3.8%) *Speech*: 2 (7.69%)
7.	EXTRA-CRANIAL MOVEMENTS	10 (38.46%)
	Shoulder shrugging: 2 (7.69%)	Twisting movements of upper limbs and neck: 1 (3.8%)
	Upper limb tremors only: 3 (11.53%)	Neck dystonia: 1 (3.8%)
	Upper limb tremors with lower limb tremors: 1 (3.8%)	Functional hemiparesis with Numbness: 1 (3.8%)
	Writing problems: 1 (3.8%)	
8.	PRECIPITATING FACTORS	
	Emotional stress: 8 (30.76%)	Road traffic accident: 1 (3.8%)
	Financial constraints: 6 (23.07%)	Gastric upset: 1 (3.8%)
	Job pressure and work-related stress: 2 (7.69%)	Claustrophobia: 1 (3.8%)
	Refusal to go to boarding school: 1 (3.8%)	Keen observation of his own face while shaving: 1 (3.8%)
	Brother went missing from home: 1 (3.8%)	
9.	ASSOCIATED ILLNESSSES	
	*Headache*: 4 (15.38%)	
	***Neuropsychiatric disturbances:* 7 (26.92%)** Unspecified: (3.8%) Anxiety: 3 (11.53%) Depression: 2 (7.69%) Feelings of loneliness and isolation: 1 (3.8%)	***Organic illnesses;* 4 (15.38%)** Subdural hematoma: 1 (3.8%) Seizure disorder: 1 (3.8%) Organic cervical dystonia: 1 (3.8%) Intracranial mass: 1 (3.8%)
10.	OUTCOME	
	Improved: 9 (34.61%)	Did not improve: 4 (15.38%)
	Partially improved: 13 (50%)	

**Table 2:** Clinical characteristics of 26 patients of cranial functional movement disorders.

Case #	Age (years)/Sex (M: Male, F: Female)	Duration of illness	Onset-abrupt/gradual	Predominant cranial movement disorder	Additional cranial movement disorder	Additional extracranial movement disorder	Precipitating factor	Associated illness	Outcome

Case 1	34/F	5 days	Abrupt	Left hemifacial spasm	Jaw deviation to the left side	–	Fight with husband	–	Improved
Case 2	24/F	3 months	Gradual	Variable hemifacial spasm	Deviation of mouth to one side	Tremulousness of right upper and lower limbs	Family conflicts	–	Partially improved
Case 3	16/F	3 years	Gradual	Right hemifacial spasm	–	–	Family stress	Headache (Migraine)	Partially improved
Case 4	11/F	5 months	Gradual	Bilateral ptosis (Right > Left)	–	–	Refusal to go to boarding school	–	Partially improved
Case 5	24/F	One and half year	Gradual	Chin tremor	Twitching of left angle of mouth	Bilateral postural hand tremors with distractibility	Anxiety regarding her imminent marriage	–	Partially improved
Case 6	30/F	3 days	Abrupt	Tongue dystonia	–	–	Death of father-in-law who was the sole earning member of family and financial constraints	–	Improved
Case 7	16/F	4 months	Gradual	Palatal tremor	–	–	Brother went missing from home, sick uncle at home	Left occipital ganglioglioma	Partially improved
Case 8	83/F	4 months	Gradual	Lip-smacking movements with abnormal noises (orofacial dyskinesias)	–	Bilateral shoulder shrugging	Emotional stress as she was forced to stay away from her son	–	Improved
Case 9	40/M	2 days	Abrupt	Stuttering and effortful speech	–	Bilateral hand tremors that are variable, distractibility present	Financial loss due to short-circuit at home	–	Improved
Case 10	40/F	1 day	Abrupt	Child-like prosody	–	Left-sided functional hemiparesis with numbness	Financial constraints	Anxiety, depression, headaches	Partially improved
Case 11	35/M	6 years	Gradual	Eyebrow dyskinesia	Abnormal tongue movements	Bilateral hand tremors with distractibility	Financial issues	–	Improved
Case 12	25/M	3 years	Gradual	Orofacial dyskinesia	–	Twisting movements of upper limb and neck	–	Anxiety, loneliness, avoids friends	Partially improved
Case 13	62/M	15 days	Gradual	Orofacial dyskinesia	–	Bilateral shoulder shrugging	–	Subdural hematoma	Partially improved
Case 14	21/M	One month	Gradual	Orofacial dyskinesia	–	–	–	Neuropsychiatric disturbances	Partially improved
Case 15	37/M	One year	Gradual	Bruxism	–	–	Job pressure	–	Partially improved
Case 16	65/F	2 days	Abrupt	Mouth-opening dystonia	–	–	Claustrophobia during MRI	Organic cervical dystonia	Improved
Case 17	31/M	5 months	Gradual	Tongue tremor	–	–	Looking at himself in the mirror while shaving	–	Improved
Case 18	41/F	One year	Gradual	Oromandibular dyskinesia	Jaw deviation to the left side	–	Husband stays away in Dubai	–	Partially improved
Case 19	48/M	2–1/2 years	Gradual	Blepharospasm	Ocular tics for 1 year	Writing problems for 10 days	Work-related pressure	Anxiety	Partially improved
Case 20	25/F	1 day	Abrupt	Tongue dystonia	–	–	Gastric upset	–	Improved
Case 21	50/F	5 months	Gradual	Right hemifacial spasm	–	–		Depression	Not improved
Case 22	40/M	4 months	Gradual	Facial dyskinesia	–	–	Financial constraints	–	Not improved
Case 23	20/F	4 days	Abrupt	Jaw spasm	–	–	Road traffic accident 4 days back	–	Not improved
Case 24	13/M	3 years	Gradual	Bilateral eyelid myoclonus	–	–	Fight with a friend	Seizure disorder for 5 years, headache for 3 days	Partially improved
Case 25	16/F	3 years	Gradual	Tongue protrusion	–	Functional neck dystonia	Financial constraints	–	Improved
Case 26	36/F	2 years	Gradual	Orofacial dyskinesia	–	–	Fight with husband	Headache	Not improved

### Demographics

In our cohort of 159 patients of FMDs, CFMDs were present in 26 patients (16.35%). The majority of our CFMDs patients were females (16; 61.53%) and their mean ± [SD] age was 33.96 ± [16.94] years (range 11 to 83 years). There were 5 children (age of onset <18 years) and 3 elderly (age of onset >60 years) patients in our cohort. The duration of illness ranged from one day to 6 years (mean± SD: 402.03 ±534.97 days).

### Clinical features

Of the 26 patients diagnosed with CFMDs, 24 (92.30%) met the Fahn-Williams diagnostic criteria for the documented, and 2 (7.69%) for the clinically established. We have provided the videos of 9 patients highlighting the wide spectrum of phenomenology in our CFMDs cohort (See video). The onset of illness was abrupt in 26.92% (7/26) and gradual in 73.07% (19/26) of patients. Most of the patients had symptoms pertaining to either one half or the whole of the face with the majority having orofacial dyskinesias (n = 5; 19.23%) and hemifacial spasm (n = 4; 15.38%). Out of the 5 cases of orofacial dyskinesias, 3 were men and 2 were women. All the patients with hemifacial spasm were females and as compared to organic hemifacial spasm, it was seen to be fixed instead of clonic in Case 1 and inconsistent in their location (alternating from side to side) in Case 2 (See videos). Amongst the 4 (15.38%) cases with movements in and around the eyes, we had one case each of bilateral eye ptosis (Case 4: See video), blepharospasm, bilateral ‘painful’ eyelid myoclonus and eyebrow dyskinesia in the form of repetitive frontalis ‘overactivity’. The tongue involvement was seen in 4 patients (15.38%) in the form of dystonia (n = 3) and tremor (n = 1), and 3 of them were females. We came across several unusual phenomenologies including bruxism, chin tremor, mouth-opening dystonia, oromandibular dyskinesia, and jaw spasm each in one patient. Case 7 (See video) was that of a young female with a palatal tremor that disappeared on using a tongue depressor. There were 2 cases of functional speech and voice disorders both of which were abrupt in onset: a male with stuttering voice (Case 9: See video) and one female with childlike prosody along with left-sided functional hemiparesis with numbness (Case 10). Six of the 26 (23.07%) patients also had additional craniofacial movements along with the primary movement disorder.

### Distractibility, variability, and entrainment

Clinical incongruency and inconsistency are the hallmarks of the FMDs and are necessary for the diagnosis to be certain. Seventeen (65.38%) of our patients had variability in their symptoms, 10 (38.46%) had distractibility and 3 (11.53%) had entrainment.

### Additional extracranial movements

Ten (38.46%) of our patients also had concomitant functional movements involving the body regions other than the craniofacial areas and what is remarkable is that 9 (90%) of them were related to the shoulder and upper limbs: 2 cases had bilateral shoulder-shrugging movements, 4 cases had bilateral upper limb tremors, one had twisting movements of upper limbs, one had writing difficulty for 10 days, and one patient also had left-sided hemiparesis with numbness of the same regions [Hoover’s sign was positive] with acute speech disturbance. Lower limb tremors and functional neck dystonia were present in one patient each.

### Precipitating factors

Twenty-two (84.61%) out of 26 of our patients had a well-defined stressor for these abnormal movements. Family conflicts and emotional stress were the most common precipitating factors present in 30.76% (n = 8) of patients and interestingly, all cases with these stressors were females. In male patients, stress related to financial constraints was the most common precipitating factor. Two of the patients had physical inciting factors such as gastric upset and road traffic accidents. The children and young adults mostly had factors related to school stress and feelings of anxiety and social stress.

### Associated illnesses

Nine of our patients had associated illnesses and 5 of them had neuropsychiatric issues in the form of anxiety and depression and 4 patients had associated headaches. Organic illnesses in the form of subdural hematoma, cervical dystonia, seizure disorder, and intracranial space-occupying lesion were present in 4 patients that couldn’t be attributable to the movement disorder the case presented with.

### Outcome

At the 3-months follow-up, 9 (34.61%) out of 26 patients had improved whereas 4 (15.38%) of them failed to show any improvement whatsoever. The overwhelming majority of the patients (13/26; 50%) had only partial recovery of their symptoms with a variable course.

### Summary of the cranial functional movement disorders cases reported in the literature

Out of 311 articles that we retrieved for our systematic review, 25 were found to be relevant (Figure 1; supplementary online table [7,8,9,11,12,13,14,17,20,21,26,27,28,37,38,39,40,41,42,43,44,45,46,47,48]. Of the total number of 434 patients in all these studies, 191 (44%) were females and 48 (11%) were males. For the remaining 195 (44.93%) patients, gender was not specified. The majority of the patients were found to have some form of facial or eye involvement. The movement related to eyes was mostly unspecified (n = 188) with others being that of blepharospasm (n = 3) and pseudoptosis (n = 1). Out of all the abnormal facial movements, pure hemifacial spasm (37/152; 24.34%), and facial dystonia (17/152; 11.18%) were the most common. Other involved regions were jaw (n = 119), speech and voice (n = 107), platysma (n = 89), tongue (n = 79), palatal or lip (n = 33) and head (n = 10). The phenomenologies of 12 patients were not specified in the respective studies. For a total of 434 patients, 793 phenomenologies were described which translates into the fact that most of these patients had multiple functional movements involving various parts of the body. Clearly mentioned overlaps were observed in about 60 (16%) of 375 patients. The detailed phenomenologies of patients with overlaps were reported by Factor et al [11] [head tremor with eyelid fluttering (n = 1), blepharospasm with dysphonia (n = 1), and head tremor with intermittent tongue protrusion (n = 1)], Chung et al [12] [(speech abnormality with some facial abnormal movement (n = 5)], Kamble et al [13] [abnormal head movements + tremors (n = 2), mutism + gait abnormality (n = 2), facial dystonia + mutism (n = 2), palatal myoclonus + astasia abasia + facial dystonia (n = 1) and facial dystonia + tremors (n = 2)] and Stone et al [14] [one case with right “pseudoptosis” with contraction of orbicularis oculis and oris which gave an impression of right facial weakness with unilateral photophobia in the right eye]. In a study of the 101 patients with functional abnormalities, Teodoro et al reported that effortful facial expression and other responses (e.g. frowning, grimacing, effortful breathing) in 26.7% of patients and/or excessive blinking in 38.6% of patients predominated, with at least one of them being present in 40.6% (41/101) patients [15].

## Discussion

This is a case series of 26 patients with various CFMDs collected over 2 years. The prevalence of FMDs is two to three times higher in women than in men and this is well evident in our study in which the majority of the patients were young females (16/26; 61.53%) [7].

### Eyes

Ocular or peri-ocular movements seem to be common when it comes to FMDs as is evident from two other case series (Fasano et al with 51% patients out of 61 cases and Stone et al with 90% patients out of 41 cases) with a large number of cases with at least some form of eye involvement [7,8]. We had 4 patients who had abnormal movements related to the eyes: one patient each of bilateral ptosis, blepharospasm, eyebrow dyskinesia, and bilateral ‘painful’ eyelid myoclonus. In a large series of 101 patients clinical examination triggered facial and eye movement disorders in 46% of patients, but, this phenomenon was not observed in any of our patients [15]. In the same vein, Stone et al in 2010 reported a case with recurrent episodes of right “pseudoptosis” with contraction of orbicularis oculis and oris which gave an impression of right facial weakness with unilateral photophobia in the right eye [14]. Our patients did not have any concomitant facial abnormal movements. Convergence spasm has been previously reported as the most frequent functional eye movement disorder and it was seen in as many as 69% of FMDs cases in a study reporting 13 patients [9,16]. However, we did not come across any patient with convergence spasm.

### Face

Out of our 26 patients, 10 of them had varied facial involvement among which 5 had orofacial dyskinesias, 4 were that of hemifacial spasm and one patient had pure facial dyskinesia. In 2001, Tan et al reported 5 patients (all females) and in 2016, Baizabal-Carvallo et al reported 15 patients with facial movement [17,18]. Consistent with the literature, all our 4 hemifacial spasm patients were females. The onset of the movements was gradual in all of them barring one in whom the onset was abrupt. The clues to psychogenicity in these patients were movements mostly involved in the lower half of the face, were variable and distractible. In one case it was seen to be fixed instead of clonic and in another movement were inconsistent in their location (alternating from side to side) [4]. None of our patients reported facial spasms during sleep (said to be present in up to 80% of organic hemifacial spasm or any worsening of spasms during voluntary facial contractions (seen in up to 39% of hemifacial spasm patients); most patients had lower-face involvement at onset in contrast to the isolated lid involvement typically present at the onset in organic hemifacial spasm [19].

### Stomatognathic system: jaws, lips, tongue, teeth, and associated soft tissues

Several case series have shown the very relevant involvement of this system when it comes to facial functional movement disorders (FFMDs) [7,8]. The most common phenotype is tonic jaw deviation accompanying ipsilateral downward and lateral lip pulling, seen in 84.3% of patients with FFMDs involving the craniofacial region but we did not have any such patient in our series which perhaps highlights the varied presentations of FMDs in different geographic areas [7]. Yoshida et al in their very recent study, recorded 58 patients with FMDs in the stomatognathic system in which functional dystonia phenotype was observed in 44.8% and 27.6% of their patients showed very fast repeated jaw and/or lingual movements and 72.4% of their cases were females [20]. This data is similar to our study in which 77.7% (7/9) patients were women. What is significant and should be noted is that 50% of the cases of the Yoshida et al study reported pain whereas none of our patients reported it [20]. Also, none of our patients had precipitating factors such as dental (44.8%) or physical trauma (12.1%) that were the commonest causes seen in the study published by Yoshida et al [20]. Whereas abnormal jaw and tongue movements have been earlier reported in various case series, none of them describing bruxism as our study has [7,8,20]. Functional palatal and lip tremors have been reported but functional chin tremor is rare and we report one such case (Case 5; see video) [20,21]. We also report one young girl (Case 7; see video) with a psychogenic palatal tremor that was accompanied by bilateral ear clicking. Biller et al in 2013 proposed to segregate essential palatal tremor into 3 clinical overlapping variants: psychogenic, un-voluntary or secondary essential, and voluntary essential palatal tremor [22]. Also, as per the literature, the female gender and the presence of bilateral ear clicking in our patient is consistent with psychogenicity [23]. Our patient did not have acute upper respiratory tract infection as a precipitating factor as reported in other studies [21,24]. Platysma overactivity has been commonly reported previously to co-exist with FFMDs (61% in Fasano et al, 85% in Stone et al) but only 7.6% (2/26) of our patients were found to have it [7,8].

### Speech

Various studies have reported that between 16.5% and 53% of FMDs patients have a comorbid functional abnormality in speech or voice [11,25,26,27,28]. While dysphonia, stuttering, and prosodic abnormalities are common among functional voice and speech disorders (FSVDs), it is important to realize that any aspect of speech or phonation may be affected [12]. Acquired organic stuttering often presents with dysarthria, aphasia, or apraxia of speech; the absence of these features is a red flag for a functional etiology [12]. One female in our cohort had abrupt onset functional hemiparesis with concomitant functional child-like prosody and 3 out of the 6 cases reported by Chung et al who had prosodic disturbances were females as well [12]. We report one male patient (Case 9: See video) with functional stuttering (FS) and this is consistent with the literature that says that FS is equally prevalent among males and females, contrasting with the 3:1 male: female ratio observed in patients with organic stuttering [29]. In contrast with organic motor speech disorders (OMSDs), patients with FSVDs often exhibit inconsistencies and considerable variability in their speech or phonation and their symptoms may alter considerably with distraction or suggestibility [30]. Patients with FSVDs may also exhibit struggle behaviour resulting in exaggerated facial movements, including marked facial grimacing, lip pursing, eye blinking or contraction of the periorbital, lower facial muscles, or platysma during an attempted speech [12]. Patients complaining of weakness may paradoxically exhibit speech with a strained quality or exaggerated facial posturing that is inconsistent with their complaint of weakness [31]. Also, the course of FSVDs can be highly variable and in one conversation many qualitative changes in the character of the dysphonia or severity of the symptoms can occur and symptom reversibility is one of the most important clinical features of FSVDs [32]. It is crucial to diagnose these disorders as deficits in patients with FSVDs also have a greater potential for reversibility than those in OMSDs patients and while speech therapy rarely provides improvement for patients with OMSDs, various studies have documented that a course of speech therapy can be quite effective for many patients with FSVDs [12,33].

### Extra-craniofacial psychogenic movements

The co-occurrence of different functional disorders is well known [34]. 38.46% (10/26) of our patients had concomitant functional movements involving the body regions other than the craniofacial areas and 90% (9/10) of them were related to the shoulder and upper limbs. This seems to be consistent with the literature as extra-facial contractions involved upper limb in 30% of the 61 patients in the series by Fasano et al and 78% patients out of the 41 cases evaluated by Stone et al had evidence of functional limb weakness and 22% had limb dystonia [7,8].

### Precipitating factors

Since the advent of modern psychiatry, a correlation between the experience of emotional trauma and psychogenic symptoms has been postulated [35]. Kletenik et al pointed out that trauma and adverse life events are important risk factors for developing FMDs and they have also reported that the higher prevalence of FMDs in women is likely related to both higher rates of sexual abuse against women and the impact of sexual abuse in women [36]. But in our Indian society, there seems to be a significant impact of culture, ethnicities, and other psychosocial variations on the precipitating factors. We differ from the western literature when it comes to precipitating factors for FMDs as, in our country, even today, there are germane concerns regarding a lack of proper communication between the physician and the patient, especially women, who do not usually come out with complaints of sexual abuse or marital conflicts to avoid running the risk of social embarrassment. Seven out of sixteen women (43.75%) in our study, however, did cite familial disharmony on careful probing and three of them reported financial difficulties. 5 out of 10 men (50%) had been facing financial constraints and work-related stress, whereas, in children, family separation, school-related performance pressure, tiffs with friends, and absence of a sibling were common propellants in our study. Physical stressors seem to be relatively less common with one case with a prior history of a road traffic accident and one with a preceding bout of severe gastric upset. Two of our patients had interesting risk factors with one case developing the FMD after an episode of claustrophobia and one case who developed tongue tremor while keenly observing himself in the mirror during shaving.

### Associated illnesses

Eleven out of twenty-six (42.30%) of our patients had some form of associated illness. Four of them reported headaches (4/26; 15.38%) which is similar to the study conducted by Stone et al in which migraine triggered functional facial movement disorders in 17% of the patients [8] Neuropsychiatric disturbances ruled the roost with seven (26.92%; 7/26) of our patients reporting it either in the form of anxiety, depression, feelings of loneliness and isolation with one of them not being specified. Depression and headache were also common in the series by Fasano et al [7]. Other organic clinical entities that were found to co-exist in 15.38% (4/26) patients. Examples include subdural hematoma, organic cervical dystonia, seizure disorder, and an intracranial mass was found in one patient each and this observation is important as it emphasizes the fact that quite often organic and functional pathologies can overlap and that the presence of an organic pathology does not rule out a functional element or vice versa.

**Video:** **Cranial functional movement disorder patients (cases 1–9): Abnormal movements, variability and distractibility are seen in different patients**. **Case 1:** A 34-year old lady with an abrupt onset fixed left-hemifacial spasm of 5 days duration after a squabble with her husband. **Case 2:** A 25-year old female with gradual onset variable hemifacial spasm of either side of her face for the last 3 months with bilateral tremulousness of her upper and lower limbs as well. She cited family conflicts as a stress factor. **Case 3:** A 16-year old female with gradual onset hemifacial spasm of either side of her face for the last 3 years which is variable and distractible while finger tapping and when her face is being touched by a pen. **Case 4:** An 11-year old girl presented with bilateral (right > left) intermittent ptosis for 5 months that started to develop after she was being forced to go to the boarding school. On finger tapping with the right hand, there is entrainment in the eyelid blinking. **Case 5:** A 24-year old female has chin tremors of one-and-a-half-year duration. She reported anxiety regarding her impending marriage. On finger tapping from the left hand, her chin tremor decreases significantly. **Case 6:** A 25-year old female developed abrupt onset abnormal movements of the tongue for 3 days. She had significant difficulty in speaking **(Segment 1)**. Her symptoms improved significantly within a week following counselling **(Segment 2)**. **Case 7:** A 16-year girl who presented with a palatal tremor of 4 months duration that was variable and could be completely suppressed on manually depressing the tongue with a spatula. **Case 8:** A 83-year old woman developed 2 episodes of abnormal lip-smacking movements with bizarre noises lasting for about 5 days each for 4 months that was precipitated due to emotional stress **(Segment 1)**. Her symptoms used to subside while engaged in conversation **(Segment 2)**. **Case 9:** A 40-year old male with abrupt onset stuttering of speech of 2 days duration. On examination, bilateral hand tremor is also present which is variable and distracted while finger tapping from the left hand.

### Outcome

Little is known about the course and the outcome in CFMDs patients. At the end of three months, 34.61% of our patients had improved with no relapses, 15.38% did not show any improvement and an overwhelming majority of 50% patients showed partial improvement only with a variable course. In the study reported by Fasano et al, the course was stable in 53%, variable in 33%, and had diurnal fluctuations in one-fifth.

Certain limitations should be kept in mind before the interpretation of the results of our study. The major limitation was represented by its retrospective chart review design, which has certain inherent errors such as missing data, recall bias, and follow-up details. Further, our patients did not follow a standard protocol-based treatment which may have affected the outcome. There were some inadvertent limitations in the systematic literature review also which was markedly affected by the incomplete data provided in various studies. We were not able to figure out the genders of a huge majority of cases and phenomenologies of all the patients could not be well delineated as many authors presented their data in the form of percentages only and we could only roughly calculate the probable patient number [7,8].

Despite all these limitations, our study has reported some important observations regarding CFMDs patients. First, females were more likely to have CFMDs similar to other FMDs and facial involvement was more likely to occur than other cranial musculatures. Second, we have reported some unusual phenomenologies like chin tremor, ptosis, eyebrow dyskinesia, and bruxism. Third, functional movements involving the body regions other than the craniofacial areas were present in one third (38.46%) of our patients and 90% of them were related to the shoulder and upper limbs. Fourth, precipitating factors were present in 84.61% of the patients and associated illnesses were present in 42.30% of the patients. The most common precipitating factors in females were emotional and familial stress, whereas financial stress was the most common in males. Fifth, one-third of our patients had a good outcome, whereas half of the patients had a variable course.

## Conclusion

Our study has provided additional data regarding CFMDs from India, once again reinforcing the fact that FMDs are universally present and that it is important for neurologists to be aware of signs and symptoms suggestive of FMD. The CFMDs should be considered if there is fixed unilateral facial contraction supported by normal neurological examination. There is some geographical variation regarding the phenomenology also, like convergence eye movement and lower lip commonly described in CFMDs patients were not seen in our cohort. An early diagnosis based on the phenomenology is important as it will avoid unnecessary diagnostic investigations and allow early intervention with the appropriate therapy.

## Additional File

The additional file for this article can be found as follows:

10.5334/tohm.352.s1 Supplementary Online Table. Review of literature of cranial functional movement disorders: Details of published cases.
