# Supplementary material for: Cranial Functional Movement Disorders: A Case Series with Literature Review
Source: Tremor Other Hyperkinet Mov (N Y). 2020 Aug 25;10:33. doi: 10.5334/tohm.352 (PMC7485402; doi:10.5334/tohm.352)
Supplement: Supplementary Online Table. — Review of literature of cranial functional movement disorders: Details of published cases. [file tohm-10-1-352-s1.pdf]

Supplementary Online Table: Review of literature of cranial functional movement disorders: Details of published cases.

Reference number	Author/ year	Total number of cases	Number of CFMD cases	Details of CFMD	Precipitating factor	Outcome
1.	Williams 1995[37]	131 PMD cases	4 FFMD	Blepharospasm or other facial movements	NA	NA
2.	Factor 1995[11]	1 FSVD case out of 28 FMD cases	1 female	Case 28:<br>Slow halting voice<br>(38/F)	NA	NA
		8 FFMD cases out of 28 FMD cases	5 females, 3 males	Functional facial movement disorders		
				Case 9: Blepharospasm<br>(50/F)	NA	Unresolved
				Case 12:<br>Head tremor<br>(58/F)	Unemployment	NA
				Case 14:<br>Head tremor<br>(39/F)	Personal injury	Resolved with psychological counselling
				Case 16:<br>Head tremor with eyelid fluttering<br>(59/M)	NA	Unresolved
				Case 17:<br>Jerking head movements<br>(48/M)	NA	Resolved

				Case 25:<br>Blepharospasm with dysphonia<br>(42/F)	Disability	Resolved with improvement in psychosis
				Case 27:<br>Head tremor with intermittent tongue protrusion<br>(47/M)	Delusion of possession	NA
				Case 28:<br>Tongue movements<br>(38/F)	Litigation; after taking erroneous prescription drug	NA
3.	Lang 1995[38]	14 cases of functional Parkinsonism	1 FSVD	Soft voice, speech bizarre	NA	NA
4.	Tan 2001[17]	210 hemifacial spasm cases	5 females with functional hemi-facial spasm	Hemi-facial spasm		
				Case 1<br>(33/F)	Stress, fatigue, anxiety	Remission at 2 months
				Case 2<br>(45/F)	Stress and anxiety	Remission at 3 months, lost to follow up
				Case 3<br>(27/F)	Stress and anxiety	Remission and relapse
				Case 4<br>(42/F)	Fatigue	Remission

				Case 5<br>(26/F)	Stress, fatigue, anxiety	Remission and relapse
5.	Hinson 2006[39]	88 FMD	25 FSVD	Speech severity was rated as minimal to mild	NA	NA
6.	Ferrara 2008[40]	54 FMD	4 FSVD	Disrupted speech	NA	NA
7.	Ertan 2009[41]	49 FMD	4 FSVD	Bizarre speech	NA	NA
8.	Stone 2010[14]	1	40/F	Recurrent episodes of right "pseudoptosis" with contraction of orbicularis oculis and oris which gave an impression of right facial weakness with unilateral photophobia in the right eye	NA	NA
9.	Kranick 2011[42]	64 FMD	8 FSVD	No description of speech disorder	NA	NA
10.	Yaltho 2011[43]	215 cases of HFS	16 psychogenic out of which 15 women	Psychogenic facial movements: spasm, dystonia, tics or myoclonus	NA	NA
11.	Czarnecki 2012[44]	60 FMD	7 FSVD	No description of speech disorder	NA	NA
12.	Saifee 2012[26]	26 FMD	14 FSVD	Difficulty speaking/slurred speech	NA	NA

13.	Stamelou 2012[21]	7 psychogenic out of 17 cases of palatal tremors	Case 1: 30/F	Palatal tremor(PT)	Viral labyrinthitis(3 weeks)	Symptoms better after psychotherapy BoNT: improvement after 3 days; further BoNT treatments no clear benefit;2 weeks treatment with10 mg amitriptyline, clear improvement
			Case 2: 34/F	PT	Tinnitus (same time)	Pregabalin, levetiracetam, clonazepam: no benefit; diazepam: better; BoNT refused
			Case 3: 33/F	PT	Flu-like illness (2 days)	BoNT not tried
			Case 4: 40/F	PT	Sore throat, right sided otitis(same time)	Clonazepam, no benefit, BoNT refused; cognitive behavioral therapy declined
			Case 5: 47/F	PT	Flu(1 week)	BoNT improvement
			Case 6: 28/F	PT	Endoscopy for nausea(2 weeks)	BoNT refused
			Case 7: 28/M	PT	Vomiting, vertex headache (same time)	Benzehxol,baclofen,diltiazem,levodopa,clonazepam:nobenefit;fluoxetine,paroxetine,BoNT:improvement
14.	Fasano 2012[7]	61 patients of functional facial movement disorders	56 (91.8%) females		Psychological stress-22<br><br>Physical trauma-9<br><br>Peripheral facial injury-8	All but 6 subjects received a variety of medical and non-pharmacological treatments without any benefit (56%) or worsening (20%). Aside from complete remissions

					Pain-3<br><br>Another disease-5	(following a treatment or not), 20% of treated patients improved after treatment [BoNT] at therapeutic doses was effective in 5 cases, antidepressants in 3, antiepileptics in 2, and psychotherapy in 1.
15.	Batla 2013[45]	18 FMD	4	Blepharospasm:3; Facial spasm: 1	NA	NA
16.	Erro 2014[46]	1	Female	Fixed tongue dystonia with acute right Bell's palsy	NA	NA
17.	Baizabal-Carvallo 2015[27]	182 FMD	30 FSVD	Stuttering, speech arrests, hypophonia, dysphonia, Foreign Accent Syndrome	NA	NA
18.	Colosimo 2015[47]	1	29/M	Right eye and left lower face tonic spasms	NA	Low dose botulinum toxin treatment as well as a placebo injection of saline, administered subcutaneously in the left hemiface, led to a partial improvement in the symptoms. The clinical picture subsequently resolved spontaneously some months later
19.	Gözke 2016[48]	1	44-year old female	Psychogenic facial dystonia	NA	Resolution of symptoms within 1 week with alprazolam
20.	Kamble 2016[13]	15 out of 73 FMD patients	2 females, 1 male	Abnormal head movements(3)	NA	NA
			2 females, 1 male	Facial dystonia(3)	NA	NA
			1 female	Abnormal facial movements(1)	NA	NA

			2 males	Abnormal head movements + tremors(2)	NA	NA
			2 males	Mutism + gait abnormality (2)	NA	NA
			2 males	Facial dystonia + mutism (2)	NA	NA
			1 female	Palatal myoclonus + astasia-abasia + facial dystonia(1)	NA	NA
			1 male	Facial dystonia + tremors(1)	NA	NA
21.	Baizabal-Carvallo 2017[28]	15 out of 184 FMD patients	14 females, 4 males; mean age of onset- 33 ± 13.5 years	Hemifacial spasm	Trauma was reported by four patients; severe stressors by five; medications by one; history of sexual abuse by one and migraines by one patient	A single patient with psychogenic HFS was treated with BoNT with mild benefit.<br><br>All patients with psychogenic HFS were referred for stress management or psychological counselling with moderate benefit.
22.	Chung 2018[12]	6		Functional speech and voice disorders with/without functional facial movement disorders		
				Case 1:<br>29/F<br>Childlike prosody and intervals of slow, deliberate speech with long pauses prior to speech initiation with intermittent facial grimacing and functional lower face dystonia	NA	NA

				Case 2:<br>59/F<br>Intermittent childlike speech prosody resulting from forward pursing of the lips and forward positioning of the tongue with an unusually high-pitched voice along with a downward retraction of her lower face during speech, simulating a central facial droop	NA	NA
				Case 3:<br>46/M<br>Dysfluent speech with frequent pauses and intermittent stuttering along with distractible facial grimacing and excessive platysmal contraction	NA	NA
				Case 4:<br>38/F<br>Acute onset dysphonic speech accompanied by distractible struggle behavior, with facial grimacing and repeated pauses scattered throughout her spontaneous speech	NA	NA
				Case 5:<br>67/F<br>Sudden onset of stuttering speech and aphonia	NA	NA
				Case 6:<br>57/F<br>Slow, deliberate speech with articulation and grammatical errors, and spoke with childlike prosody	NA	NA
24.	Stone 2018[8]	41 FFMD cases	Mean age: 44 years ,	Eye 90%<br>Mouth 90%<br>Unilateral Platysma Contraction 85%	Migraine triggered functional facial movement disorders in 17%	NA

			81% females	Jaw Deviation 12%<br>Tongue deviation 17%<br>Convergence Spasm 22%<br>Dysphonia 12%		
23.	Teodo2019[15]	101	-	*Before formal examination:*<br>1. blurred vision-12.9%<br>2. double vision-5%<br>3. oscillatory eye movement-3% (including one case of voluntary nystagmus)<br>4. increased eye blinking-7.9%	NA	NA
				After formal examination: 45.5%<br>1. Saccades were abnormal in 42.6% and smooth pursuit movement in 36.6%	Formal eye examination	NA
				Effortful facial expression and other responses (e.g. frowning, grimacing, effortful breathing) (26.7%) and/or excessive blinking (38.6%) predominated, with at least one of them being present in 40.6% patients	NA	NA
25.	Yoshida et 2020[20]	58	Mean age: 46.2 years<br><br>Sex (women 42 (72.4%), men 16 (27.6%)	Jaw deviation 43 (74.1%)<br><br>Jaw closing 29 (50.0%)<br><br>Lip (pulling or deviation) 20 (34.5%)<br><br>Tongue (protrusion or deviation) 18 (31.0%)<br><br>Cervical (platysma or sternocleidmastoid) 17 (29.3%)<br><br>Jaw opening 13 (22.4%)	Dental treatments 26 (44.8%)<br><br>Tooth extraction 14 (24.1%)<br><br>Bridge 4 (6.9%)<br><br>Crown 3 (5.2%)<br><br>Dental operation 3 (5.2%)<br><br>Implant treatment 1 (1.7%)<br><br>Orthodontic treatment 1 (1.7%)	Reported improvements:<br>Pharmacotherapy 34 (58.6%)<br>Muscle afferent block therapy 19 (32.8%)<br>Botulinum toxin therapy 28 (48.3%)<br>Sensory trick splint therapy 16 (27.6%)<br><br>Surgical therapy (coronoidotomy) 1 (1.7%)

				Around mouth 12(20.7%)<br><br>Around eye 10 (17.2%)<br><br>Facial 8 (13.8%)<br><br>Tremor (lip or palate) 5 (8.6%)<br><br>Jaw protrusion 2 (3.4%)<br><br>Others 7 (12.1%)	Physical trauma 7 (12.1%)<br><br>Trauma 4 (6.9%)<br><br>Operation 2 (3.4%)<br>Dislocation 1 (1.7%)<br><br>Psychological stress 3 (5.2%)<br><br>Another disease 1 (1.7%)	

Footnote: FFMD- Functional facial movement disorder, FMD-Functional movement disorder, FSVD-Functional speech and voice disorder, HFS-Hemifacial spasm, BoNT-Botulinum neurotoxin, PT-Palatal tremor, NA- Data is not available
